# Correlation of histopathological features and clinical findings in pterygium

**DOI:** 10.6026/973206300211443

**Published:** 2025-06-30

**Authors:** Hithesh I., Sangeetha T., Hemalatha A., Vrushabh M.

**Affiliations:** 1 Department of Ophthalmology, Sri Devaraj Urs Medical College, Kolar, Karnataka, India - 563101; 2 Department of Pathology, Sri Devaraj Urs Medical college, Tamaka, Kolar, Karnataka, India

**Keywords:** Epithelial hyperplasia, ocular surface, squamous, neoplasia, vascularity

## Abstract

The correlations between clinical signs and histo-pathological features are of interest. Hence, a prospective study of 48 primary pterygia cases examined. Most patients were female, with common symptoms including redness, cosmetic concerns and astigmatism. Histology showed squamous hyperplasia, inflammation and vascular changes. Clinical redness significantly correlated with epithelial hyperplasia and lymphocytic infiltration. Data suggest some pterygia may mimic ocular surface squamous neoplasia, impacting diagnosis and treatment.

## Background:

Pterygium is a common ocular disorder characterised by the fibrovascular sub-epithelial ingrowth of elastotic degenerative bulbar conjunctiva over the limbus onto the cornea with a prevalence of 13.2% across plains, hilly and coastal regions of India [1]. Contributing factors include chronic UV radiation, hot climates and environmental elements like dust and wind, especially in equatorial regions [2]. Although typically benign, pterygium can significantly affect quality of life due to cosmetic concerns, irritation, refractive errors due to astigmatism or visual axis obstruction in advanced stages. Surgical excision is the main treatment for symptomatic pterygium. While simple excision has a high recurrence rate (15-75%), conjunctival auto-grafting and free flap surgery reduce recurrence to around 6%. Outcomes depend on the technique, use of mitomycin C and pterygium characteristics [3]. Ocular surface squamous neoplasia (OSSN), which shares risk factors with pterygium-especially UV exposure-occurs in about 0.3% of pterygium cases [4]. Diagnosing OSSN in this context is difficult due to overlapping features, though OSSN is more aggressive and may undergo malignant transformation. Histo-pathological examination remains the gold standard for diagnosing. Therefore, it is of interest to report the correlation of histopathological features with the clinical findings of pterygium.

## Materials and Methods:

This prospective hospital based study was conducted on 48 patients with pterygium, in the department of Ophthalmology from September to December 2024 after approval from the Institutional Ethics Committee and written informed consent from patients. Data included age, gender, occupation, duration, cosmetic and visual complaints, clinical signs and the histopathologic results of excised tissue from all patients who underwent pterygium excision with conjunctival limbal autograft transplantation using a standardized surgical technique. Subjects were excluded if they had a history of prior medical treatment for pterygium such as topical corticosteroids or non-steroidal anti-inflammatory drugs, previous conjunctival surgery, conjunctival cicatricial disorders, systemic autoimmune diseases or dry eye disease. Preoperative assessment included a comprehensive ophthalmic evaluation consisting of slit-lamp biomicroscopic examination and grading of pterygium [5], grading of vascular congestion and redness based on clinical appearance [6] (Table 1 - see PDF). Followsed by assessment of best-corrected visual acuity (BCVA) by Snellen chart and astigmatism by autorefractometer. Excised tissue specimens were immediately fixed in 10% buffered formalin. The samples were subsequently processed, embedded in paraffin, sectioned and stained with hematoxylin and eosin (H and E). Histopathological evaluation included analysis of both epithelial and stromal parameters. Data is analysed using SPSS version 22 software with p-value of <0.05 was considered statistically significant.

## Results:

A total of 48 patients diagnosed with pterygium were included in the study. With a mean age of 57.5 + 13.3 (range 30-88 years), Majority of them were females 79.2% (38), with males comprising 20.8% (10). Bilateral pterygium was prevalent, observed in 62.5% (30) of cases, while 37.5% (18) had unilateral involvement. In terms of refractive changes, 60.4% (29) had astigmatism > 2.5 D, compared to 39.5% (19) who had < 2.5 D. Analysis of pterygium grading revealed that Grade II was the most frequent, identified in 43.7% (21) Grade I in 41.67% (20) and Grade III in 14.5% (7), indicating fewer cases of advanced disease (Table 2 - see PDF). The most commonly reported symptom was cosmesis, noted by 70% followed by foreign body (FB) sensation, in 60% of cases. In contrast, redness and irritation were less frequently observed, affecting 11% and 9% of patients, respectively. Table 3 (see PDF) summarizes the distribution and statistical relevance of epithelial and stromal histopathological changes in pterygium. Among epithelial changes, hyperplasia was most common (74.3%), followed by dysplasia (50%) and metaplasia (36.4%), with a significant overall association (p = 0.046). Stromal changes showed high vascularity (92%, p = 0.001) and frequent occurrence of both fibrosis and inflammation (69.7% each), though only fibrosis was statistically significant (p = 0.001), while inflammation was not (p = 0.272). Table 4 (see PDF) summarizes the correlation between histopathological changes and clinical grades of pterygium. Inflammation was most frequent in Grade II (45.5%), followed by Grade I (42.4%) and Grade III (12.1%), with a significant difference among grades (P = 0.032). Vascularity was present in Grades I (52%) and II (48%) but absent in Grade III (P = 0.001). Fibrosis was highest in Grade II (65.2%), moderate in Grade III (26.1%) and lowest in Grade I (8.7%), also showing statistical significance (P = 0.001). Dysplasia occurred only in Grades I and II (one case each), with no significant association (P = 0.346). Hyperplasia and metaplasia were most common in Grade II (51.4% and 72.7%, respectively) and least in Grade III and I.

## Discussion:

This study evaluated the correlation between histopathological changes and clinical findings of pterygium in a cohort of 48 patients. The demographic data are in line with previous study which reported a similar mean age and less pronounced female predominance (55%) [7]. Conversely, other studies demonstrated a male predominance, likely due to increased outdoor exposure and UV radiation, a well-established risk factor for pterygium [8, 9]. Astigmatism >2.5D was observed in 60.4% of patients, indicating significant refractive impact. This is consistent with the findings of other who reported that the majority of pterygium cases induced astigmatism exceeding 2D [10, 11]. The pterygium horizontal length and thickness were moderately to strongly correlated with astigmatism while the vertical length showed no or just mild correlation with the corneal astigmatism [12]. In the present study, cosmesis (70%) and foreign body (FB) sensation (60%) were the most commonly reported symptoms, while redness (11%) and irritation (9%) were less frequently observed comparable with other studies [13, 14-15]. Collectively, these studies confirm that cosmetic and mechanical symptoms predominate, whereas inflammatory symptoms are comparatively infrequent. The histopathological findings are consistent with previous studies that have also identified epithelial hyperplasia and dysplasia as key histopathological features in pterygium (Table 5 - see PDF). The predominance of hyperplasia and vascular changes in both the epithelial and stromal layers underscores the chronic nature of the condition. These results also confirm the consistency of epithelial hyperplasia, fibrosis and vascular proliferation as prominent features of pterygium, suggesting a multifactorial etiology involving UV exposure and tissue remodelling processes. Recent research on pterygium pathology and its histopathological features often highlights the same trends observed in the current study (Table 4 - see PDF), though there are variations across studies in terms of exact percentages or grading criteria. Although pterygium is traditionally classified as a benign lesion, it exhibits several characteristics commonly associated with malignancy. Sun *et al.* studied ocular surface squamous neoplasia encompassing mild, moderate and severe epithelial dysplasia, as well as carcinoma in situ identified in 14 out of 105 (13.3%) surgically excised pterygium tissue samples [18]. In a series of 149 pterygium cases, mild to moderate dysplasia was identified in 31 (21%), a feature potentially linked to OSSN. Additionally, melanocytic hyperplasia was observed in 47 (32%) samples, which may indicate the presence of primary acquired melanosis (PAM). Dysplasia is noted in low frequencies across studies and is often considered a risk factor for malignant transformation. Hyperplasia and Metaplasia, a transformation of one type of epithelium to another is noted in more severe pterygium cases, as epithelium tries to adapt to the stress of the condition [19]. Similarly, Chui *et al.* examined the prevalence of coexisting ocular surface pathologies in patients with pterygium and reported epithelial dysplasia of varying severity in approximately 5% of cases [20]. A significant increase in inflammation and vascularity is noted in higher-grade pterygia (Grade II) compared to lower-grade (Grade I). Fibrosis increases in higher-grade pterygia, a common finding across various studies. In the table, Grade II shows the highest percentage of fibrosis (65.2%), indicating a higher degree of tissue remodelling and scarring compared to lower-grade pterygia supported other studies [21]. Present study witnessed features of ocular surface squamous neoplasia in one case. The association between these two is the common factor of UV exposure (ophthalmohelioses) that results in overexpression of p53, whereas the role of human papillomavirus is unclear. Section studied shows stratified squamous epithelium with few goblet cells. Focal areas show moderate dysplasia, congested and dilated vessels in the sub epithelium.

## Conclusion:

Clinical conjunctival hyperaemia in pterygium is significantly associated with key histopathological features and may mimic ocular surface squamous neoplasia, complicating diagnosis. Hence, routine histo-pathological evaluation of excised pterygium is recommended to detect OSSN and guide management.
